# Preventing Recurrent Laryngeal Nerve Injury During Transhiatal Esophagectomy Through Targeted Cervical Dissection: A Case Report

**DOI:** 10.7759/cureus.101288

**Published:** 2026-01-11

**Authors:** Luis Munoz-Andrade, Diego A Morales-Cisneros, Erick Zambrano-Franco, Danna V Auria-Aspiazu, Guido Panchana-Coello

**Affiliations:** 1 Surgery, Medical Center Munoz and Andrade, Quito, ECU; 2 Surgery, Instituto Oncológico Nacional Dr. Juan Tanca Marengo, Sociedad de Lucha Contra el Cancer (SOLCA), Guayaquil, ECU; 3 Surgery, Universidad de Especialidades Espíritu Santo, Guayaquil, ECU

**Keywords:** case report, cervical dissection, esophageal cancer, nerve injury prevention, recurrent laryngeal nerve, transhiatal esophagectomy

## Abstract

Injury to the recurrent laryngeal nerve (RLN) is a well-recognized complication of esophagectomy and is associated with significant postoperative morbidity, including dysphonia, aspiration, and pulmonary complications. The risk is particularly relevant during transhiatal esophagectomy, where limited mediastinal visualization increases the likelihood of traction-related nerve injury. We report a case of a 68-year-old male with distal esophageal carcinoma who underwent transhiatal esophagectomy following neoadjuvant therapy, in whom a targeted cervical dissection strategy was employed to achieve early identification and preservation of the left RLN. Key technical elements included lateralization of the thyroid lobe, selective ligation of small collateral vessels, traction-free cervical mobilization of the esophagus, and coordinated bidirectional cervical-abdominal dissection to minimize mediastinal strain. The gastric conduit was delivered through the posterior mediastinum, and a hand-sewn cervical esophagogastric anastomosis was completed without intraoperative complications. Postoperative laryngoscopic evaluation confirmed normal vocal cord mobility, and the patient experienced no dysphonia, aspiration, or respiratory events during recovery. This case highlights a potentially reproducible, anatomy-based cervical approach for RLN preservation during transhiatal esophagectomy that can be applied in resource-limited settings to reduce functional morbidity.

## Introduction

Injury to the recurrent laryngeal nerve (RLN) remains one of the most significant functional complications following esophagectomy, with reported incidences ranging from 5% to over 30%, depending on the surgical approach, extent of dissection, and use of neoadjuvant therapy [1,2]. RLN palsy is associated with substantial postoperative morbidity, including dysphonia, impaired airway protection, aspiration, pneumonia, prolonged hospitalization, and diminished quality of life, making its prevention a critical objective in esophageal cancer surgery [3].

Among the available surgical approaches, transhiatal esophagectomy continues to be widely performed, particularly in patients with distal esophageal tumors and in centers where thoracoscopic or robotic platforms are not routinely available [4]. However, the blind or semi-blind nature of mediastinal dissection inherent to this technique increases the risk of RLN injury, primarily due to traction, compression, or thermal damage during cervical mobilization of the esophagus [5]. This risk is further amplified in patients who have received neoadjuvant therapy, in whom fibrosis, tissue edema, and distorted anatomical planes complicate safe dissection and nerve identification [6].

In addition to unilateral injury, bilateral RLN palsy represents a particularly severe complication, often requiring prolonged airway support and significantly increasing postoperative morbidity, underscoring the importance of preventive strategies during esophageal surgery [7]. Previous studies have demonstrated that early identification and deliberate preservation of the RLN, rather than reliance on blind avoidance, are associated with lower rates of both transient and permanent nerve palsy [8].

Although intraoperative nerve monitoring has been shown to reduce the incidence of RLN injury during esophagectomy, its routine use is limited by cost, technical complexity, and institutional resources, particularly in low- and middle-income settings [9]. Consequently, there remains a clear need for reproducible, anatomy-based surgical strategies that allow effective RLN preservation without dependence on advanced technology [9,10].

To clearly position the contribution of this report, it is important to emphasize that the novelty of the described strategy does not lie in RLN identification alone, which is a well-established principle. Rather, this case highlights a structured, stepwise cervical dissection protocol specifically adapted to transhiatal esophagectomy without intraoperative nerve monitoring. The distinctive elements include the following: (1) a predefined cervical sequence prioritizing early thyroid lobe lateralization before esophageal mobilization; (2) selective low-thermal or cold vascular control to minimize traction and thermal spread near the nerve; (3) deliberate traction-free cervical handling of the esophagus; and (4) coordinated bidirectional cervical-abdominal dissection to reduce axial mediastinal tension during blind mobilization. A brief summary of these key technical steps is provided to facilitate reproducibility and accessibility, particularly for surgeons practicing in resource-limited settings.

In this context, we present a case of transhiatal esophagectomy in which a targeted cervical dissection strategy enabled early identification and preservation of the RLN. The approach emphasizes lateralization of the thyroid lobe, selective vascular control, traction-free cervical mobilization, and coordinated bidirectional cervical-abdominal dissection to minimize mediastinal strain [10]. This case highlights a practical and resource-conscious technique that may reduce RLN-related morbidity during transhiatal esophagectomy.

## Case presentation

A 68-year-old male with a diagnosis of distal esophageal adenocarcinoma involving the mid and distal esophagus was referred for surgical management following completion of neoadjuvant chemotherapy. Clinical staging was established preoperatively based on endoscopic and imaging findings.

The patient received neoadjuvant chemotherapy with the FLOT (fluorouracil, leucovorin, oxaliplatin, and docetaxel) regimen, administered in four cycles over an eight-week period. Surgical resection was scheduled after completion of neoadjuvant therapy following multidisciplinary evaluation. No significant treatment-related toxicities affecting surgical timing, operative planning, or perioperative management were documented.

**Figure 1:**
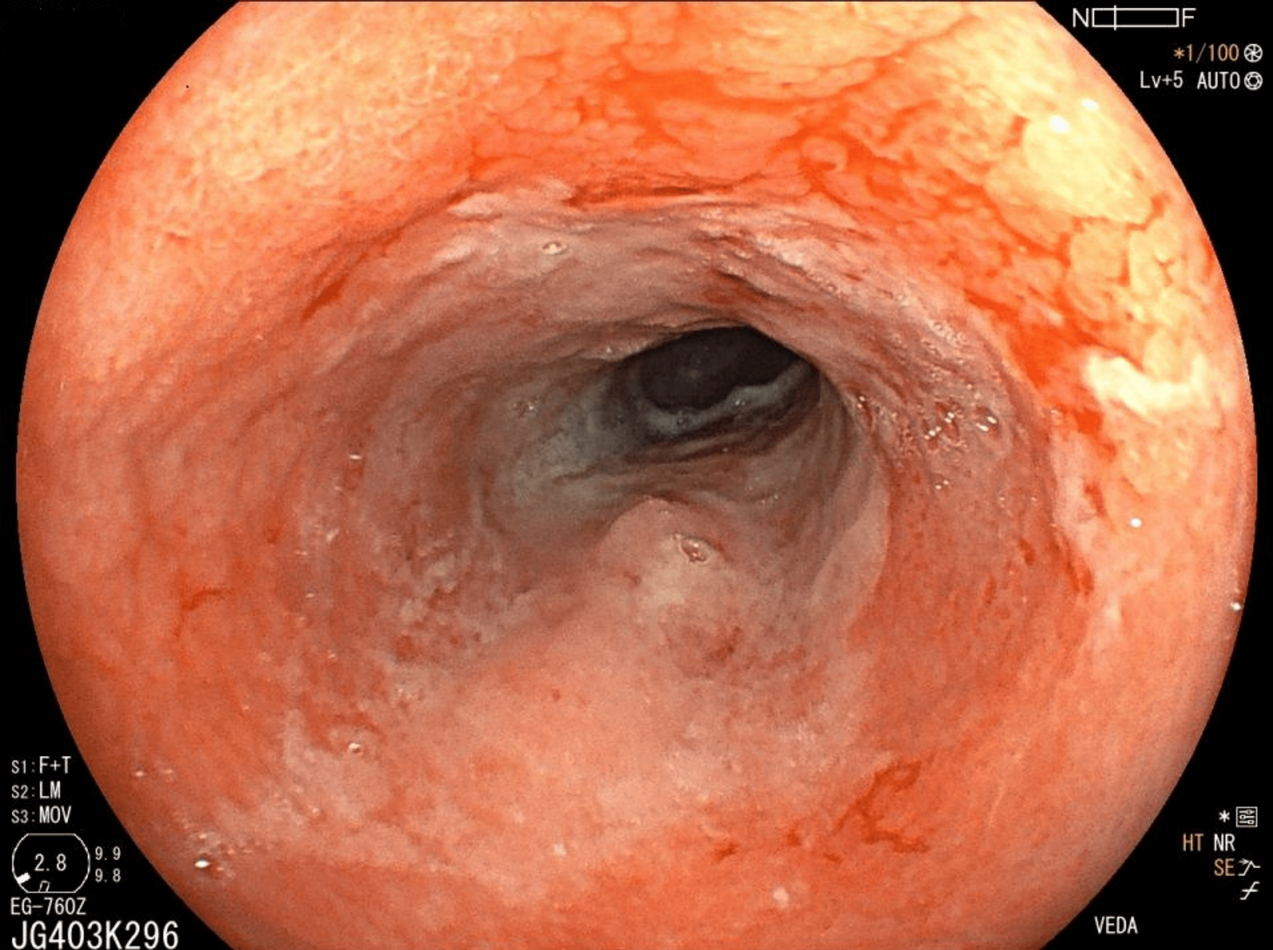
Endoscopic appearance of the distal esophagus after neoadjuvant therapy. Upper gastrointestinal endoscopy demonstrating marked tumor regression with residual mucosal irregularity and luminal narrowing at the distal esophagus prior to surgical resection.

Preoperative staging with contrast-enhanced computed tomography demonstrated a localized distal esophageal lesion without evidence of distant metastasis. Upper gastrointestinal endoscopy showed a marked response to neoadjuvant treatment, with residual mucosal irregularity and luminal narrowing at the distal esophagus (Figure 1).

The patient denied preoperative dysphonia, dyspnea, or aspiration symptoms, and physical examination revealed no cervical abnormalities. Baseline preoperative laryngoscopy was not routinely performed, as the patient had no clinical symptoms suggestive of vocal cord dysfunction.

After multidisciplinary evaluation, a transhiatal esophagectomy with cervical anastomosis was selected due to patient-related factors and institutional resource considerations. The procedure was performed under general anesthesia with the patient in the supine position. Cervical and abdominal operative fields were prepared simultaneously to allow coordinated dissection.

**Figure 2:**
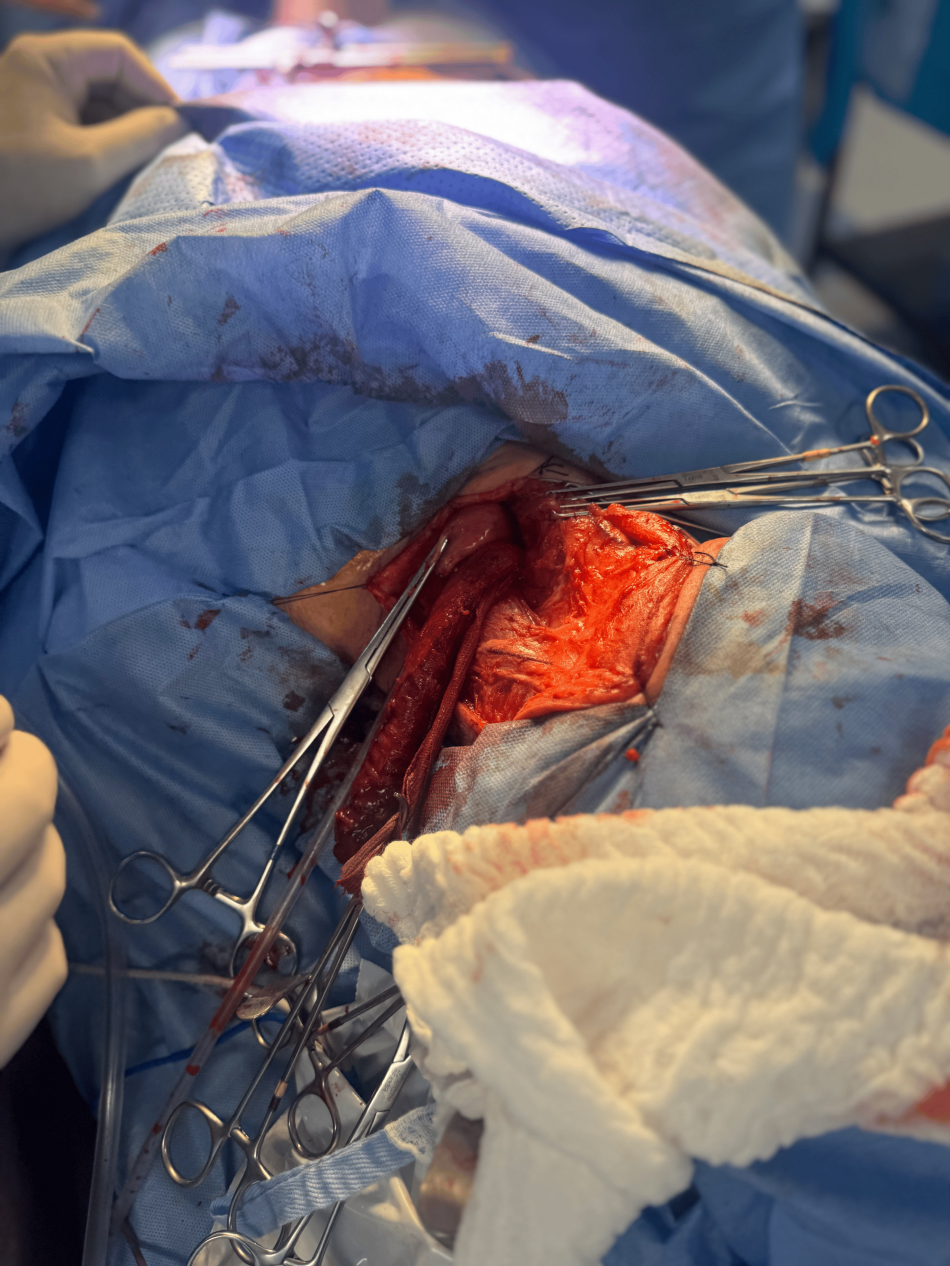
Gross surgical specimen following transhiatal esophagectomy. Resected distal esophagus obtained after completion of transhiatal esophagectomy, prior to gastric conduit reconstruction.

A left cervical incision was made along the anterior border of the sternocleidomastoid muscle. Careful dissection allowed exposure of the carotid sheath and mobilization of the left thyroid lobe. Lateralization of the thyroid gland facilitated early identification of the left RLN, which was dissected circumferentially and preserved throughout its cervical course. Selective ligation of small collateral vessels crossing the nerve was performed to avoid traction or thermal injury. All cervical maneuvers were carried out under direct visualization with strict avoidance of axial tension on the cervical esophagus (Figure 2).

While gross specimen images provide confirmation of oncologic resection, the primary educational focus of this report lies in the operative anatomy and cervical dissection strategy for RLN preservation. The figures presented should therefore be interpreted alongside the stepwise technical description, which emphasizes thyroid lobe lateralization, identification of key anatomical landmarks, selective vascular control, and traction-minimizing maneuvers during cervical esophageal mobilization.

Histopathological examination confirmed well-differentiated (G1) esophageal adenocarcinoma, measuring 5.9 × 2.5 cm, limited to the muscularis mucosae. There was no lymphovascular or perineural invasion identified, and all surgical margins were negative (R0 resection).

A total of 18 lymph nodes were examined, including 12 pericardial lymph nodes, five regional lymph nodes from the esophagectomy specimen, and one lymph node from the greater omentum, all of which were negative for metastatic disease (0/18). The final pathological stage was pT1a N0.

Attention was then directed to the abdominal phase. A midline laparotomy revealed no peritoneal or hepatic metastases. The stomach was mobilized by dividing the greater omentum and short gastric vessels while preserving the right gastroepiploic arcade to maintain conduit perfusion. A posterior mediastinal transhiatal dissection was initiated and advanced cephalad using blunt and ultrasonic dissection techniques. Due to increased mediastinal tissue stiffness following neoadjuvant therapy, a coordinated bidirectional cervical-abdominal approach was employed to minimize mediastinal traction and facilitate controlled mobilization of the esophagus.

**Figure 3:**
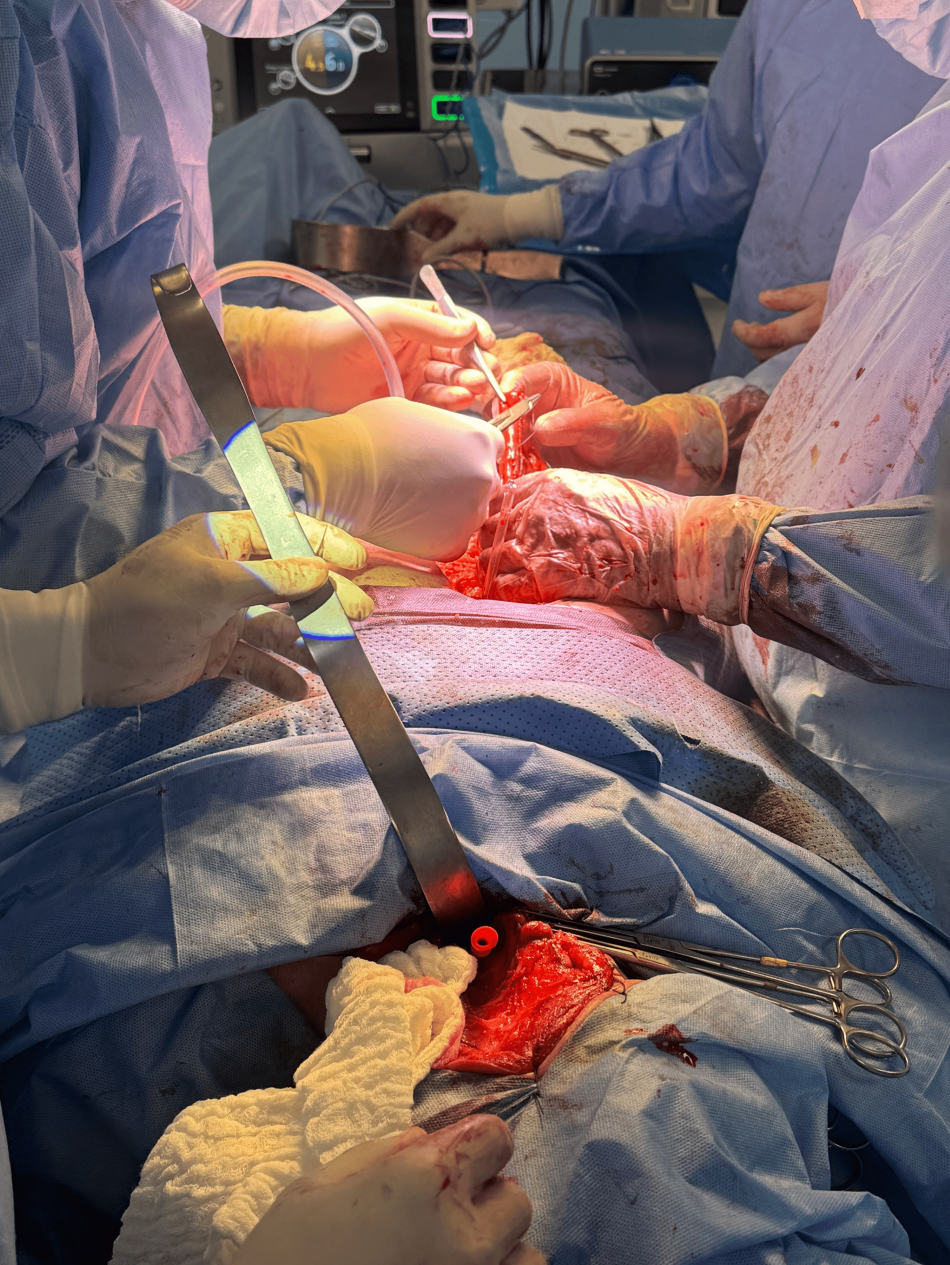
Cervical identification and preservation of the recurrent laryngeal nerve. Intraoperative cervical exposure showing lateralization of the left thyroid lobe, allowing early identification and meticulous preservation of the left recurrent laryngeal nerve during transhiatal esophagectomy.

After complete mobilization, the esophagus was delivered through the cervical incision without resistance, allowing en bloc resection of the distal esophagus (Figure 3).

**Figure 4:**
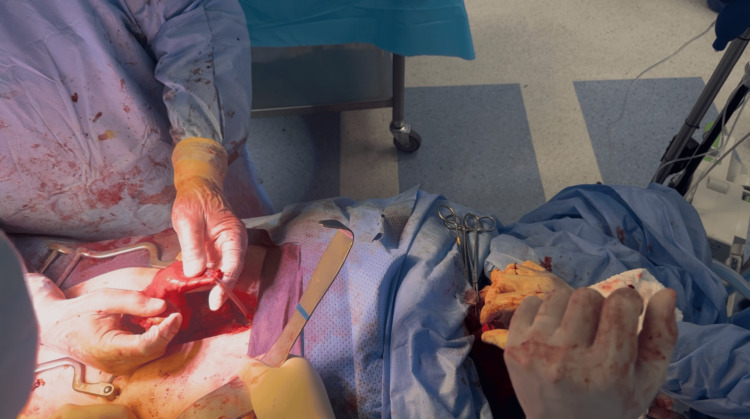
Gastric mobilization and conduit preparation. Mobilization of the stomach with division of the greater omentum and short gastric vessels while preserving the right gastroepiploic arcade to ensure adequate perfusion of the gastric conduit.

A tubular gastric conduit was then fashioned using linear staplers and reinforced with seromuscular sutures. The conduit was guided through the posterior mediastinum to the cervical field in a tension-free orientation, with careful attention to vascular integrity (Figure 4).

**Figure 5:**
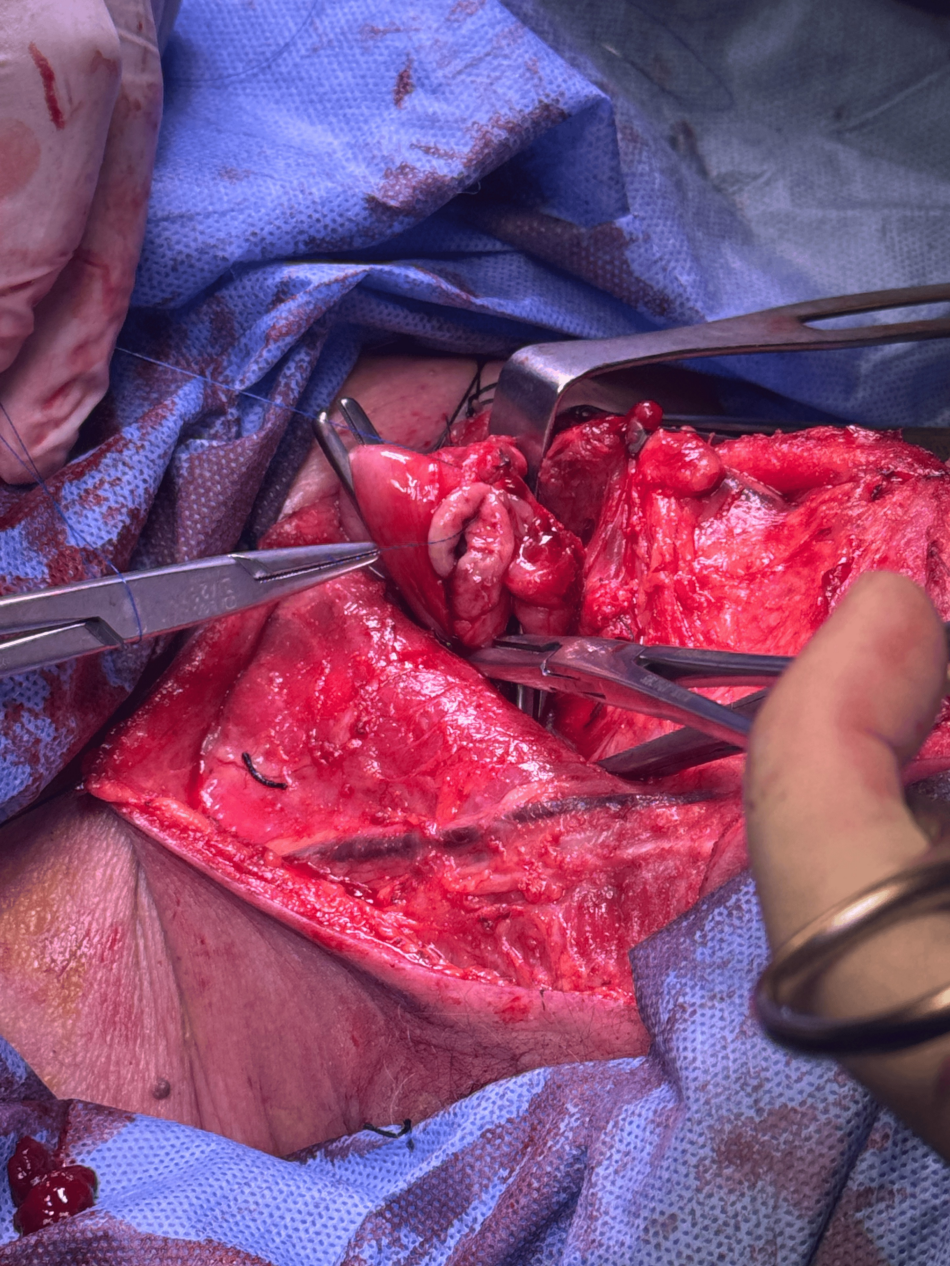
Hand-sewn cervical esophagogastric anastomosis. Two-layer end-to-side cervical esophagogastric anastomosis performed after delivery of the gastric conduit through the posterior mediastinum.

A hand-sewn, two-layer end-to-side cervical esophagogastric anastomosis was performed without technical difficulty (Figure 5).

Adequate hemostasis was confirmed, and a closed-suction drain was placed adjacent to the anastomosis prior to wound closure.

**Figure 6:**
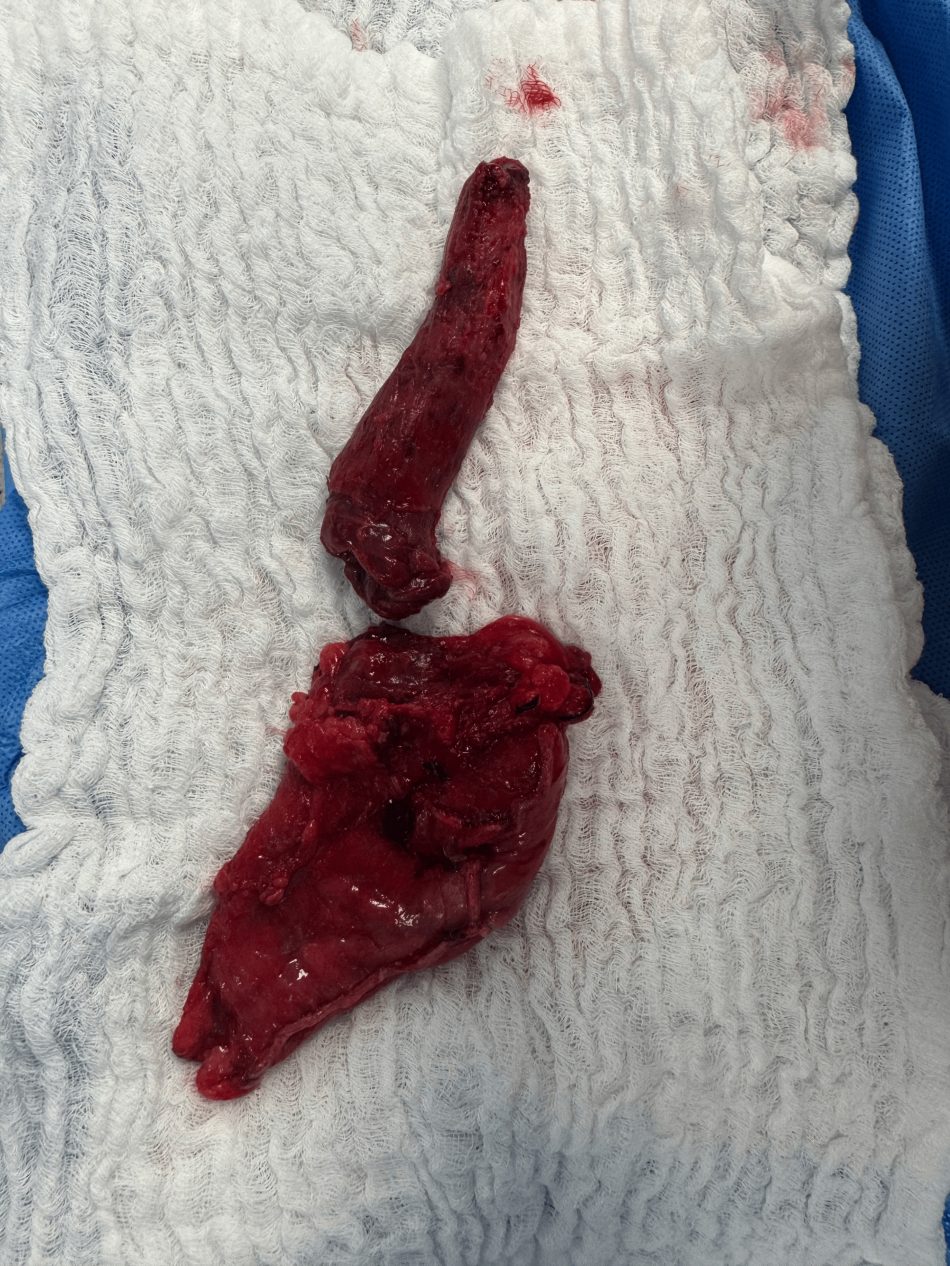
Final surgical specimen after transhiatal esophagectomy. Gross appearance of the resected distal esophagus and stomach following completion of the transhiatal esophagectomy and reconstruction.

The patient was extubated in the operating room and transferred to the intensive care unit for postoperative monitoring. Flexible laryngoscopy performed postoperatively demonstrated normal bilateral vocal cord mobility. The patient experienced no dysphonia, aspiration, or respiratory complications during recovery. A contrast swallow study showed no evidence of anastomotic leak, and oral intake was resumed progressively (Figure 6).

The postoperative course was uneventful. The operative duration and estimated blood loss were within expected ranges for transhiatal esophagectomy. The patient required a short stay in the intensive care unit and was subsequently transferred to the surgical ward. No postoperative complications were observed, and the clinical course was classified as Clavien-Dindo grade I.

Flexible laryngoscopy performed in the immediate postoperative period confirmed normal bilateral vocal cord mobility, with no evidence of RLN dysfunction. The patient remained asymptomatic, with no dysphonia, aspiration, or respiratory complaints during hospitalization and early postoperative follow-up.

A postoperative swallow study was performed prior to initiation of oral intake and demonstrated no evidence of anastomotic leak. The patient progressed to oral feeding without difficulty and was discharged in stable condition. No clinical signs of delayed vocal cord paresis or anastomotic complications were observed during early outpatient follow-up.

## Discussion

RLN injury remains one of the most impactful functional complications following esophagectomy, with consequences that extend beyond vocal impairment to include aspiration, pneumonia, prolonged hospitalization, and decreased quality of life [1,3]. Reported incidence varies widely depending on surgical approach, patient characteristics, and perioperative factors, but rates as high as 30% have been described, particularly in procedures involving extensive cervical manipulation or blind mediastinal dissection [1,2].

Transhiatal esophagectomy continues to play an important role in the surgical management of distal esophageal cancer, especially in patients with significant comorbidities or in centers where thoracoscopic or robotic platforms are not routinely available [2]. However, the inherent limitations of this approach, most notably reduced visualization of the upper mediastinum, expose the RLN to an increased risk of traction, compression, or thermal injury during cervical and mediastinal mobilization [1,4]. This risk is further accentuated following neoadjuvant therapy, where fibrosis and distorted tissue planes complicate anatomical identification and safe dissection [3,6].

Multiple studies have demonstrated that early identification of the RLN, rather than blind avoidance, is associated with lower rates of both transient and permanent nerve palsy [4,7]. Intentional exposure allows controlled dissection, selective vascular ligation, and avoidance of excessive traction, all of which are critical to nerve preservation. In the present case, lateralization of the thyroid lobe enabled early visualization of the left RLN, facilitating meticulous handling throughout the cervical phase of the procedure and minimizing the risk of injury.

The role of intraoperative nerve monitoring (IONM) in esophagectomy has been increasingly investigated, with several reports demonstrating a reduction in RLN palsy when monitoring is employed [9,10]. Nevertheless, the availability of IONM is often limited by cost, technical complexity, and institutional resources. In many low- and middle-income settings, reliance on anatomy-based surgical principles remains the primary strategy for nerve preservation. The technique described in this case illustrates that favorable functional outcomes can be achieved without IONM through careful anatomical dissection and adherence to traction-free principles.

An additional element of this case is the use of coordinated bidirectional cervical-abdominal dissection, which allowed gradual mobilization of the esophagus while minimizing mediastinal strain. Excessive axial traction has been identified as a contributing factor to RLN injury, particularly during transhiatal mobilization [3,5]. By synchronizing cervical and abdominal maneuvers, controlled advancement was achieved, reducing tension transmitted to the cervical esophagus and adjacent neural structures. Although this strategy has been variably described in the literature, it represents a simple and reproducible adjustment that may further reduce nerve-related morbidity.

The functional implications of RLN palsy following esophagectomy are substantial. Patients may develop dysphonia, ineffective cough, silent aspiration, and pneumonia, all of which negatively influence postoperative recovery and long-term outcomes [1,8]. Bilateral RLN injury, although less common, carries particularly high morbidity and may necessitate prolonged airway support or secondary interventions [5]. In the present case, preservation of normal vocal cord mobility translated into an uncomplicated postoperative course without respiratory events, supporting the clinical relevance of meticulous nerve preservation.

This case highlights a practical, anatomy-guided cervical dissection strategy that emphasizes early nerve identification, selective vascular control, traction-free mobilization, and coordinated dissection. While limited by its single-patient design, the described approach is reproducible and applicable in routine surgical practice, particularly in environments where advanced technology is not readily available. Further studies with larger cohorts are warranted to evaluate the generalizability of this technique and its impact on functional outcomes following transhiatal esophagectomy.

## Conclusions

This case illustrates an anatomy-based cervical dissection strategy aimed at minimizing RLN injury during transhiatal esophagectomy in a patient who had received neoadjuvant therapy. The absence of postoperative vocal cord dysfunction and the uneventful recovery support the feasibility of this approach in carefully selected cases. However, given the single-case nature of this report, the described strategy should be considered potentially reproducible and potentially applicable, rather than broadly generalizable. Successful implementation requires appropriate surgeon experience in cervical esophageal anatomy, meticulous traction-minimizing technique, and availability of postoperative laryngoscopic assessment.

Important limitations include the absence of a comparator group, the single-patient design, and limited follow-up duration. Further studies are needed to evaluate the reproducibility and clinical impact of this structured approach in larger patient cohorts.
